# Enhanced Adaptability to Limited Water Supply Regulated by Diethyl Aminoethyl Hexanoate (DA-6) Associated With Lipidomic Reprogramming in Two White Clover Genotypes

**DOI:** 10.3389/fpls.2022.879331

**Published:** 2022-05-20

**Authors:** Muhammad Jawad Hassan, Hongyin Qi, Bizhen Cheng, Shafiq Hussain, Yan Peng, Wei Liu, Guangyan Feng, Junming Zhao, Zhou Li

**Affiliations:** ^1^College of Grassland Science and Technology, Sichuan Agricultural University, Chengdu, China; ^2^College of Horticulture, Sichuan Agricultural University, Chengdu, China

**Keywords:** water deficit, photosynthesis, unsaturation index, glycolipids, phospholipids, sphingolipids, oxidative damage, signaling transduction

## Abstract

Membrane lipid reprogramming is one of the most important adaptive strategies in plant species under unfavorable environmental circumstances. Therefore, the present experiment was conducted to elucidate the effect of diethyl aminoethyl hexanoate (DA-6), a novel synthetic plant growth regulator, on oxidative damage, photosynthetic performance, changes in lipidomic profile, and unsaturation index of lipids in two white clover (*Trifolium repens*) cultivars (drought-sensitive “Ladino” and drought-resistant “Riverdel”) under PEG-6000-induced water-deficit stress. Results revealed that water-deficit stress significantly enhanced oxidative damage and decreased photosynthetic functions in both cultivars. However, the damage was less in Riverdel. In addition, water-deficit stress significantly decreased the relative content of monogalactocyl-diacylglycerols (MGDG), sulfoquinovosyl-diacylglycerols (SQDG), phosphatidic acisd (PA), phosphatidyl-ethanolamines (PE), phosphatidyl-glycerols (PG), phosphatidyl-serines (PS), ceramides (Cer), hexosylmonoceramides (Hex1Cer), sphingomyelins (SM), and sphingosines (Sph) in both cultivars, but a more pronounced decline was observed in Ladino. Exogenous application of DA-6 significantly increased the relative content of digalactocyl-diacylglycerols (DGDG), monogalactocyl-diacylglycerolsabstra (MGDG), sulfoquinovosyl-diacylglycerols (SQDG), phosphatidic acids (PA), phosphatidyl-ethanolamines (PE), phosphatidyl-glycerols (PG), phosphatidyl-inositols (PI), phosphatidyl-serines (PS), ceramides (Cer), hexosylmonoceramides (Hex1Cer), neutral glycosphingolipids (CerG2GNAc1), and sphingosines (Sph) in the two cultivars under water-deficit stress. DA-6-treated Riverdel exhibited a significantly higher DGDG:MGDG ratio and relative content of sphingomyelins (SM) than untreated plants in response to water deficiency. Furthermore, the DA-6-pretreated plants increased the unsaturation index of phosphatidic acids (PA) and phosphatidylinositols (PI) in Ladino, ceramides (Cer) and hexosylmonoceramides (Hex1Cer) in Riverdel, and sulfoquinovosyl-diacylglycerols (SQDG) in both cultivars under water stress. These results suggested that DA-6 regulated drought resistance in white clover could be associated with increased lipid content and reprogramming, higher DGDG:MGDG ratio, and improved unsaturation index of lipids, contributing to enhanced membrane stability, integrity, fluidity, and downstream signaling transduction.

## Introduction

Because of elevated temperatures and ever-increasing human population, depletion of available water resources is becoming a serious threat to the agricultural sector worldwide (Wang et al., 2003). Plants experience numerous distractions in their phenotypical, physiological, and metabolic levels, leading to reduced growth and productivity under water-deficient conditions (Liu et al., 2013a; Cui et al., 2017). Among physiological functions, photosynthesis occupies the core importance, and previous studies have reported that plants under water-deficit stress often experience loss of turgor, stomatal closure, a decline in carbon dioxide assimilation, chlorophyll degradation, and a significant decrease in net photosynthetic rate (Pn) (Xu et al., 2008; Soares-Cordeiro et al., 2009). When plants suffer from water-deficit stress, disequilibrium between excitation and utilization of electrons during photosynthesis results in massive production of reactive oxygen species (ROS) (e.g., OH^−^, ${\text{O}}_{2}^{-}$, and H_2_O_2_), which are responsible for enhanced electrolyte leakage and lipid peroxidation (Reddy et al., 2004; Liu et al., 2015). Many studies have shown that there exists a close relationship between photochemical efficiency of photosystem II and ROS metabolism during the photooxidation process (Van Kooten and Snel, 1990; Wang et al., 2006). Moreover, ROS impart serious oxidative damage to membranes of numerous cell organelles, metabolites, and tissues including carbohydrates, lipids, enzymes, proteins, RNA, or DNA, thus accelerating programmed cell death (Gill and Tuteja, 2010). Plant tolerance and adaption to unfavorable environmental conditions demand adequate composition and organization of cellular membranes such as alteration in lipidomic profile (Ladjal et al., 2000). Maintenance of membrane stability, fluidity, and integrity increases plants' aptitude to diverse abiotic stresses (Gasulla et al., 2013). Previous studies have reported that reprogramming of membrane lipids was an effective strategy for plants against adverse environmental stresses (Vigh et al., 2005; Degenkolbe et al., 2012).

Phospholipids (Phls) are primary constituents of extraplastidic membranes, and they not only take part in structural functions but also play a crucial role as signaling molecules in plants (Skinner et al., 2005). Bilayer-prone phosphatidylcholine (PC) and nonbilayer-prone phosphatidylethanolamine (PE) are the most abundant Phl classes in extra-chloroplast membranes associated with membrane stability and integrity (Norberg and Liljenberg, 1991; Larsson et al., 2006). In addition, extra-chloroplast membranes also contain various other Phl classes in varying quantities, such as phosphatidylinositol (PI) and phosphatidylserine (PS), performing vital roles in biosynthesis pathways of other lipid classes and stress signaling (Larsson et al., 2006; Liu et al., 2013b). Many studies have reported that the production of various signaling lipids including PI and phosphatidic acid (PA) was amplified in plants responding to environmental stresses such as high temperature, water deficiency, and osmotic stress, which regulated downstream signaling pathways contributing to physiological functions (Cowan, 2006; Munnik and Vermeer, 2010; Testerink and Munnik, 2011; Chen et al., 2018b).

Glycolipids (Glls) are another important group of lipids performing imperative functions in cell membrane stability and integrity. Among Glls, DGDG and MGDG are the chief constituents of photosystem II (PSII) and play a vital role in thylakoid membrane establishment and photochemical efficiency of PSII (Mizusawa and Wada, 2012). DGDG and PC exhibit bilayer-forming attributes, whereas MGDG and PE are prone to forming non-bilayer phases called hexagonal phases (Aronsson et al., 2008). Therefore, higher PC/PE and DGDG/MGDG ratios are believed to be important indicators of enhanced membrane stability and fluidity (Narayanan et al., 2016). The study of Chen et al. (2018a) has found that a maize (*Zea mays*) cultivar with higher DGDG content and DGDG/MGDG ratio in leaves maintained better chloroplast membrane stability and delayed leaf senescence than the other cultivar under water-deficit stress (Chen et al., 2018a). Moreover, several studies published earlier reported that a higher DGDG/MGDG ratio is associated with plant adaption to adverse environmental conditions such as water-deficit, salinity, and heat stress (Torres-Franklin et al., 2007; Wang et al., 2014; Chen et al., 2018a; Zhang et al., 2019). In addition to DGDG and MGDG, SQDG, an anionic lipid species in thylakoid membranes, is involved in the regulation of PSI and PSII under abiotic stresses (Sato, 2004; Kobayashi, 2016). Tolerant genotypes of wheat (*Triticum aestivum*) accumulated higher SQDG content than susceptible genotypes when subjected to water stress, which indicated a beneficial function of SQDG in plants under water-deficit conditions (Quartacci et al., 1995).

Sphingolipids (Spls) are an extremely complicated and diverse group of lipids that occur in a wide range of organisms such as fungi, viruses, plants, and animals (Sperling and Heinz, 2003). Spls constitute up to 40% of all lipid groups of cell membranes associated with ionic permeability and structural integrity of membrane (Markham et al., 2013). Synthesis of Spls initiates as a result of serine and palmitoyl-CoA condensation regulated by serine palmitoyltransferase in the endoplasmic reticulum (Hanada, 2003). Different types of Spls are richly available in cell membranes, including long-chain bases (LCBs), long-chain base phosphates (LCBPs), and compound sphingolipids, such as ceramide (Cer), glucosylceramide (GlcCer), glycosylinositolphosphoceramide (GIPC), and hydroxyceramide (hCer) (Markham et al., 2013). The study of Zhou et al. (2016) revealed that Spls played positive roles in resistance to chilling stress in tomato (*Solanum lycopersicum*) plants. However, potential functions and mechanisms associated with changes in Spl content and composition have not been well-understood in plants under abiotic stress, especially in response to water-deficit stress.

In the agricultural sector, diethyl aminoethyl hexanoate (DA-6), a novel synthetic plant growth regulator (PGR), has been widely utilized for many commercial crops including tomato (*Solanum lycopersicum*), cotton (*Gossypiumhirsutum*), soybean (*Glycine max*), maize (*Zea mays*), pakchoi (*Brassica rapa* subsp. *chinensis*), and peanut (*Arachishypogaea*) since the last decade because of its promising effects on improving seed germination, early seedling establishment, chlorophyll biosynthesis, photosynthetic performance, carbon and oxygen metabolism, and biomass accumulation in many plant species under favorable and unfavorable conditions (Yu et al., 2009; Jiang et al., 2012; Qi et al., 2013; Liu et al., 2019; Zhou et al., 2019; Lu et al., 2020). Regulatory mechanisms of stress tolerance induced by DA-6 have been demonstrated in different plant species. For example, the application of DA-6 alleviated the adverse effects of lead (Pb) on perennial ryegrass (*Lolium perenne*) by decreasing Pb migration and compartmentalizing Pb into the cell wall and the vacuole (He et al., 2013). DA-6 effectively detoxified cadmium (Cd) toxicity in *Amaranthus hybridus* by retaining Cd in the cell wall (Li et al., 2018). Salt-induced damage in *Cassia obtusifolia* seedlings could be mitigated by DA-6 application because of enhanced osmolyte accumulation, photosynthesis, and antioxidant defense (Zhang et al., 2016). Our recent study also demonstrated that DA-6 improved the seed germination of white clover (*Trifolium repens*) by mediation of endogenous hormones, amylolysis, and dehydrin accumulation under water stress (Hassan et al., 2021). However, how reprogramming lipid profile is linked with DA-6-induced drought resistance has not been investigated in plants so far. The objectives of this study were to investigate genotypic variations in drought resistance of white clover, which is an important cool-season leguminous plant for forage, for ground cover, and as an ornament, to determine the effect of DA-6 application on oxidative damage, osmotic adjustment, and photosynthesis, and to further elucidate how primary lipid groups (glycolipids, phospholipids, and sphingolipids) and their ratio and unsaturation levels influenced by DA-6 treatment contribute to membrane stability, integrity, and fluidity under water-deficit stress. This study will provide a deep insight into the regulatory role of DA-6 in different physiological functions and lipid metabolism conferring drought resistance in plants.

## Materials and Methods

### Plant Materials and Treatments

Seeds of two white clover genotypes (drought-sensitive “Ladino” and drought-resistant “Riverdel”) were used as planting materials. Prior to sowing, the seeds were sterilized with 75% ethanol solution for 5 min and then rinsed three times with deionized water. The seeds (0.05 g) were sown in plastic containers (24 cm in length, 20 cm in width, and 15 cm in depth), and sterilized quartz sand was utilized as a medium for growth. The plastic containers were kept under controlled conditions of 12-h photoperiod, 700-μmol photon m^−2^ s^−1^ photosynthetic active radiation, 75% relative humidity, and average temperature of 23/19°C (day/night). The seeds were watered daily with distilled water for 7 days, and Hoagland's solution (Hoagland and Arnon, 1950) was used as a nutrient source of irrigation for the next 23 days. Before the onset of water-deficit stress, 30 days-old plants were pretreated by foliar application of 1.5 mM of DA-6 solution or distilled water for 3 days so that white clover plants could absorb sufficient DA-6 or an equal amount of distilled water. Four different treatments were set up for each cultivar: (1) plants were cultivated in Hoagland's solution as the control (C); (2) plants were pretreated with DA-6 and then cultivated in Hoagland's solution (C+DA-6); (3) plants were cultivated in 17% PEG 6000 (−0.3 Mpa) Hoagland's solution as water-deficit stress (P); (4) plants were pretreated with DA-6 and then cultivated in 17% PEG 6000 Hoagland's solution (P+DA-6). The best concentration of DA-6 (1.5 mM) with the most significant effects on phenotypic changes was selected based on a preliminary experiment using the concentration of 0, 0.1, 0.2, 0.4, 0.8, 1.5, and 2 mM DA-6 solutions. Leaf samples were collected during day 12 of water-deficit stress for physiological parameters and lipidomic analysis. Each treatment consisted of 4 biological replicates, and all the treatments were laid out in a completely randomized design (CRD).

### Determination of Leaf Relative Water Content and Osmotic Potential

For the estimation of relative water content (RWC), 0.1 g of fresh leaf samples were collected and immediately weighed to obtain the fresh weight (FW). The leaf samples were then submerged in deionized water for 24 h. After being blotted dry, the saturated weight (SW) was measured. Finally, the saturated leaf samples were kept in an electric oven at 80°C for 3 days to get the dry weight (DW). RWC (%) was calculated following the formula RWC (%) = 100 × [(FW–DW)/(SW–DW)] described by Barrs and Weatherley (1962). The osmotic potential (OP) in leaves was determined following the protocols of Blum (1989). Leaf samples were excised and immersed in distilled water for 8 h to make them saturated completely. Leaves were blotted to remove surface water. Tissue samples were immediately transferred to liquid nitrogen for additional analysis. After being thawed for 30 min in an ice bath, a micropestle was used to excerpt leaf sap. The leaf sap (10 μl) was injected into an osmometer (Wescor, Logan, UT, United States) to measure the osmolality (mmol/kg). OP was calculated using the following formula: OP = –[(c) × (2.58) × (10^−3^)].

### Determination of Chlorophyll Content and Photosynthetic Parameters

To measure leaf chlorophyll (Chl) content, 0.1 g of fresh leaf samples were taken, submerged in dimethyl sulphoxide (10 ml), and kept under dark conditions for 48 h. Later, leaf extract was estimated spectrophotometrically by recording the absorbance at 645 and 663 nm. The formula illustrated by Arnon (1949) was utilized to compute Chl a, Chl b, and total Chl contents. The photochemical efficiency of PSII (Fv/Fm) and performance index on an absorption basis (PIABS, a comprehensive index for reflecting the maximum photochemical efficiency and activated photochemical reaction centers of PSII) were measured using a Chl fluorescence system (Pocket PEA, Hansatech, United Kingdom). Prior to the analyses of Fv/Fm and PIABS, leaf samples with attached leaf clips were kept under dark conditions for 30 min. Pn and water use efficiency (WUE) were recorded with a portable photosynthetic system (CIRAS-3; PP Systems, United States) that supplied 400 μl L^−1^ CO_2_ and 800 μmol photon m^−2^ red and blue lights.

### Determination of Oxidative Damage and Cell Membrane Stability

Electrolyte leakage (EL) was estimated following the procedure of Blum and Ebercon (1981) with some modifications. To estimate EL, 0.1 g of leaf samples were collected and immediately immersed in centrifuge tubes containing 45 ml of distilled water. The centrifuge tubes were kept on a mechanical shaker for 24 h, and the initial conductivity (C1) of the solution was determined. Later, these tubes were placed in an autoclave at 140°C for 30 min, and the final conductivity (C2) of the solution was measured. The EL was estimated by using the following equation: EL (%) = C1/C2 × 100%. Lipid peroxidation level was determined as change in malondialdehyde (MDA) content according to the method described by Dhindsa et al. (1981). Then, 0.1 g of fresh leaves were ground in 2 ml of 50 mM phosphate buffer saline and centrifuged at 12,000 g for 10 min and the extract was collected. The extract (0.5 ml) and a reaction solution comprising 0.5% (w/v) thiobarbituric acid (TBA) and 20% (w/v) trichloroacetic acid (TCA) were homogenized in a pellet. The mixture was kept in a boiling water bath at 95°C for 15 min and was subsequently transferred to an ice bath to cool immediately. Afterward, the reaction mixture was centrifuged at 8,000 g for 10 min, and the supernatant was collected. The absorbance value of the obtained supernatant was recorded spectrometrically at 532 and 600 nm. MDA content was computed by subtraction of OD_600_ from OD_532_.

### Lipidomic Profiling Analysis

For lipid profiling analysis, 0.3 g of fresh leaves were used for lipid extraction, separation, and identification (Vanquish UHPLC/Q Exactive Plus; Thermo Fisher). Fresh leaf samples were lyophilized to obtain a constant weight. After being crushed into a fine powder, 20 mg of the leaf samples were mixed with 300 μl of a chloroform/methanol (2:1 v/v) solution. The mixture was then placed in a rotary shaker for half an hour. Later, the reaction solution was centrifuged at 12,000 g for 20 min, and the supernatant was collected. Residues were then mixed with 300 μl of isopropanol and placed in a rotary shaker for half an hour. The supernatant was collected after the mixture was centrifuged again for 20 min at 12,000 g. The mixture of two times of supernatants was utilized for lipids analysis by using Vanquish UHPLC/Q Exactive Plus (Thermo Fisher Scientific). Data were identified and analyzed with LipidSearch 4.2 (Thermo Fisher Scientific) according to accurate mass and fragments in MS-MS. The content of total lipids was calculated based on the summation of all detected lipids. Similarly, the content of total glycolipids, total phospholipids, and total sphingolipids was calculated based on the summation of these lipids in each group. The unsaturation index of different lipid groups or lipid classes was analyzed using the following equation: (Mol% lipid × N)/100. Here, mol% represents the percentage composition of each lipid molecular species, while N shows the total number of double bonds (Su et al., 2009). The value of Ladino “C” was utilized as a standard to calculate the relative content of lipids as 1.

### Statistical Analysis

Data were assessed with Statistix 8.1 (version, 8.1. Statistix, Tallahassee, FL, United States). Significant differences between the cultivars and the treatments were estimated by one-way ANOVA in combination with the least significant difference (LSD) test at the 0.05 probability level.

## Results

### Effect of DA-6 Application on Physiological Responses of Two White Clover Cultivars Under Water-Deficit Stress

**Figure 1:**
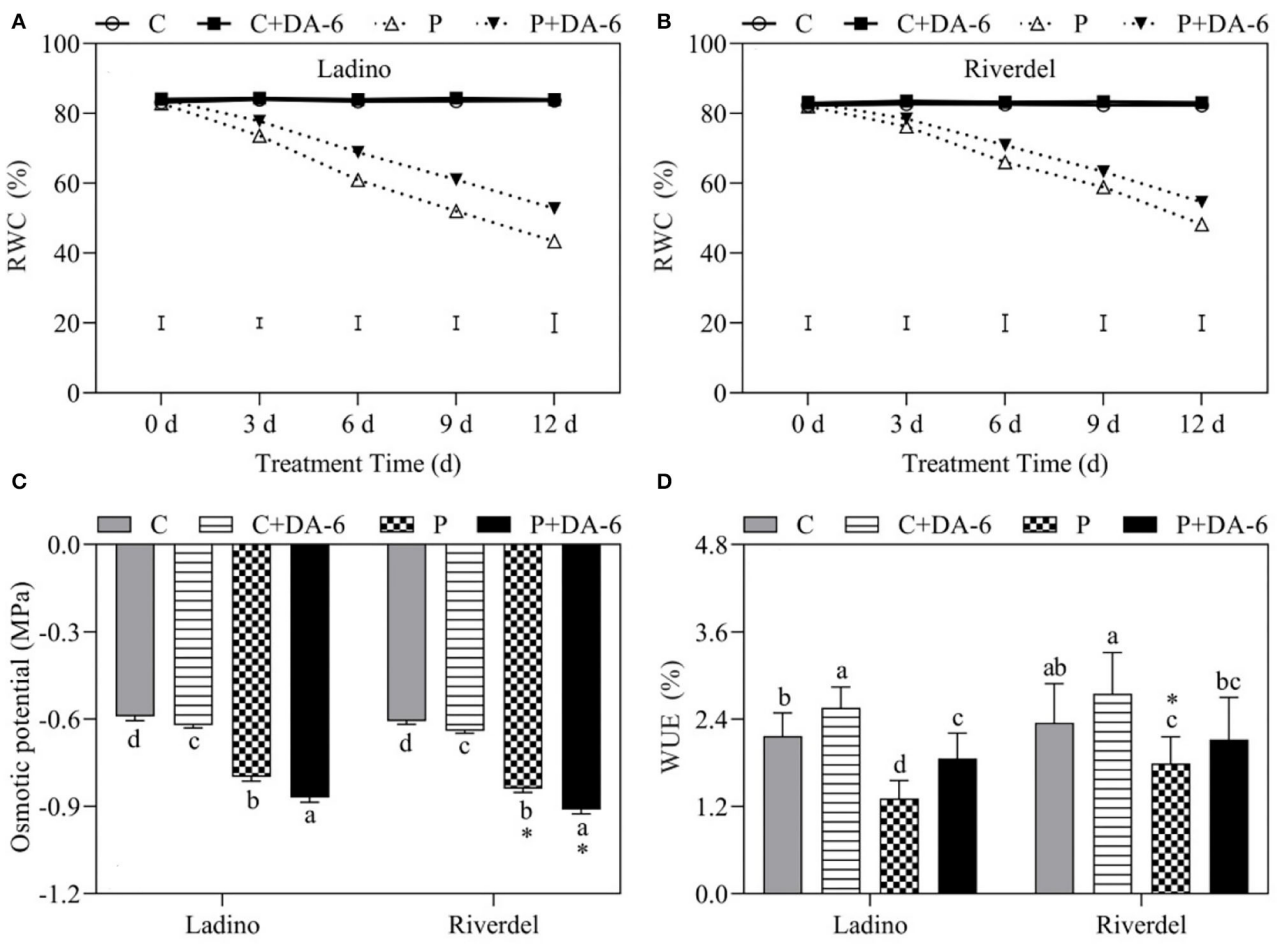
Effect of water-deficit stress and DA-6 pretreatment on **(A,B)** leaf relative water content, **(C)** osmotic potential, and **(D)** water use efficiency in the two white clover cultivars (Ladino and Riverdel). Data are mean ± standard error (*n* = 4). The vertical bars demonstrate the least significant difference (LSD) values (*p* ≤ 0.05). Different letters above or below the columns indicate significant differences between different treatments at a particular time duration under normal conditions or water stress. * represents the significant difference for a specific treatment (C, C+DA-6, P, or P+DA-6) between Ladino and Riverdel. C, control treatment; C+DA-6, control + DA-6 pretreatment; P, water-deficit stress; P+DA-6, water-deficit stress + DA-6 pretreatment.

Leaf RWC was estimated on 0, 3, 6, 9, and 12 days of drought stress to examine the water status of both cultivars (Figures 1A,B). The two cultivars did not show significant difference in leaf RWC when exposed to exogenous DA-6 application under normal conditions. Leaf RWC declined progressively as the duration of water stress was prolonged in both cultivars; however, DA-6-pretreated plants maintained significantly higher RWC under water-deficit stress (Figures 1A,B). Leaf OA and WUE were determined on day 9 of water-deficit stress when phenotypic differences were most obvious (Figures 1C,D). Leaf OA in DA-6 pretreated and untreated plants decreased significantly under water-deficit stress. In both cultivars, a minimum OP value of −0.86 and −0.9 was recorded for Ladino and Riverdel pretreated with DA-6 under water stress (Figures 1C,D). In addition, water-deficit stress decreased the WUE of both cultivars compared to respective controls. Riverdel maintained higher WUE than Ladino under water-deficient conditions. In Ladino, the exogenous application of DA-6 significantly mitigated the water deficit-induced decrease in WUE by 29.57% (Figure 1D).

**Figure 2:**
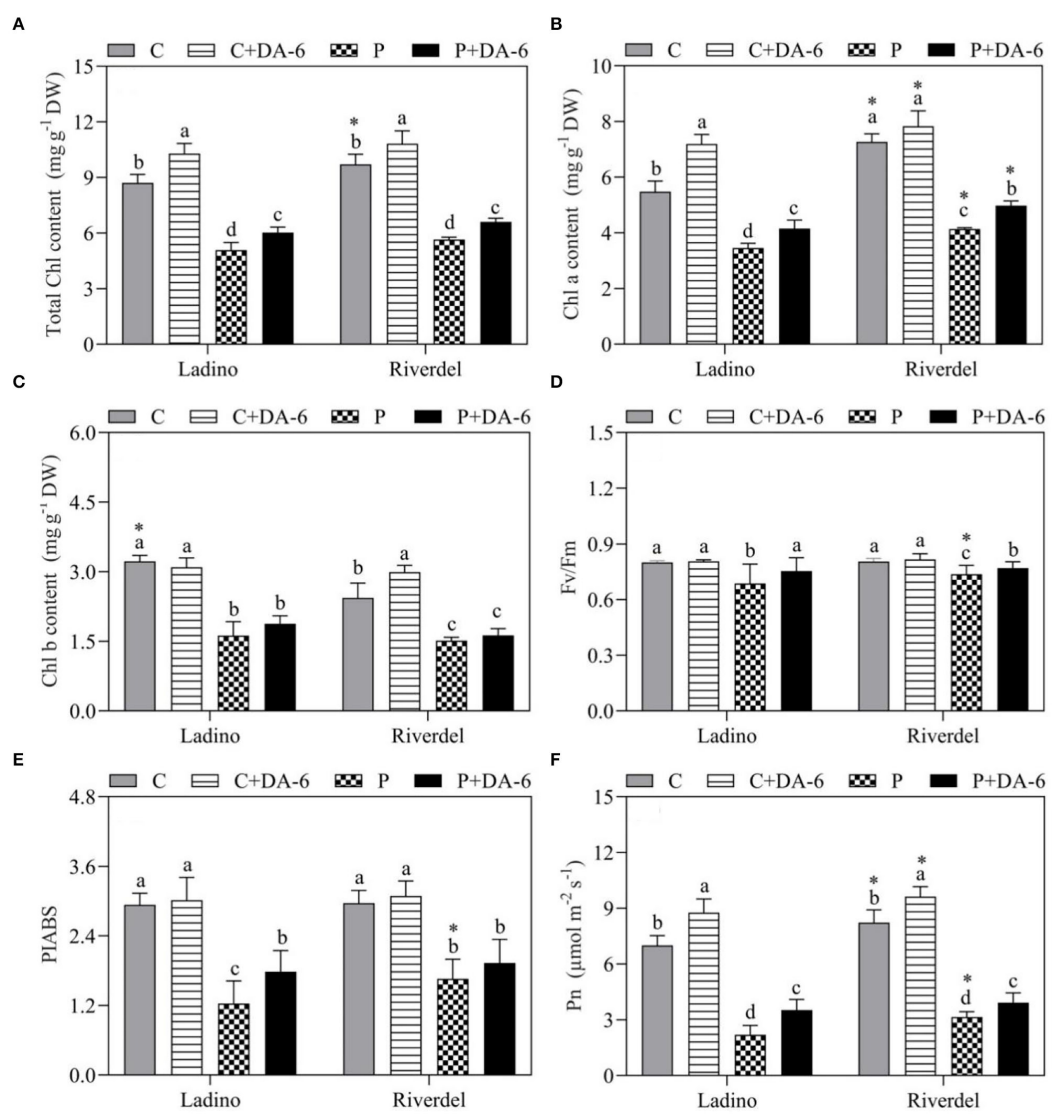
Effect of water-deficit stress and DA-6 pretreatment on **(A)** total chlorophyll content, **(B)** chlorophyll a content, **(C)** chlorophyll b content, **(D)** Fv/Fm, **(E)** PIABS, and **(F)** net photosynthetic rate in the two white clover cultivars (Ladino and Riverdel). Data are mean ± standard error (*n* = 4). The vertical bars demonstrate the LSD values (*p* ≤ 0.05). Different letters above the columns indicate significant differences between different treatments at a particular time duration under normal conditions or water stress. * represents the significant difference for a specific treatment (C, C+DA-6, P, or P+DA-6) between Ladino and Riverdel. C, control treatment; C+DA-6, control + DA-6 pretreatment; P, water-deficit stress; P+DA-6, water-deficit stress + DA-6 pretreatment.

The leaf Chl content of DA-6-treated and non-treated two white clover cultivars decreased significantly under water-deficit stress (Figure 2A). In both cultivars, the total Chl content and Chl a content of the DA-6-treated plants were significantly higher than those of the untreated plants under water-deficit stress (Figures 2A,B). In addition, the Chl b content of both drought-sensitive and -tolerant cultivars did not exhibit marked differences in response to water deficit (Figure 2C). Water-deficit stress significantly affected the Fv/Fm, PIABS, and net photosynthetic rate of the two cultivars as compared to the well-watered control (Figures 2D–F). In contrast to the plants grown under normal conditions, water-deficit stress decreased Fv/Fm by 13.8 and 8.6%, PIABS by 57.8 and 44.1%, and net photosynthetic rate by 68.1 and 61.7% in Ladino and Riverdel, respectively. Exogenous application of DA-6 on the two cultivars significantly improved Fv/Fm by 8 and 3.9% and net photosynthetic rate by 33.7 and 19.8% in Ladino or Riverdel, respectively, under water deficient conditions (Figures 2D,F). Moreover, the DA-6 treatment significantly ameliorated the PIABS in Ladino by 30.3% under water-deficit stress (Figure 2E).

**Figure 3:**
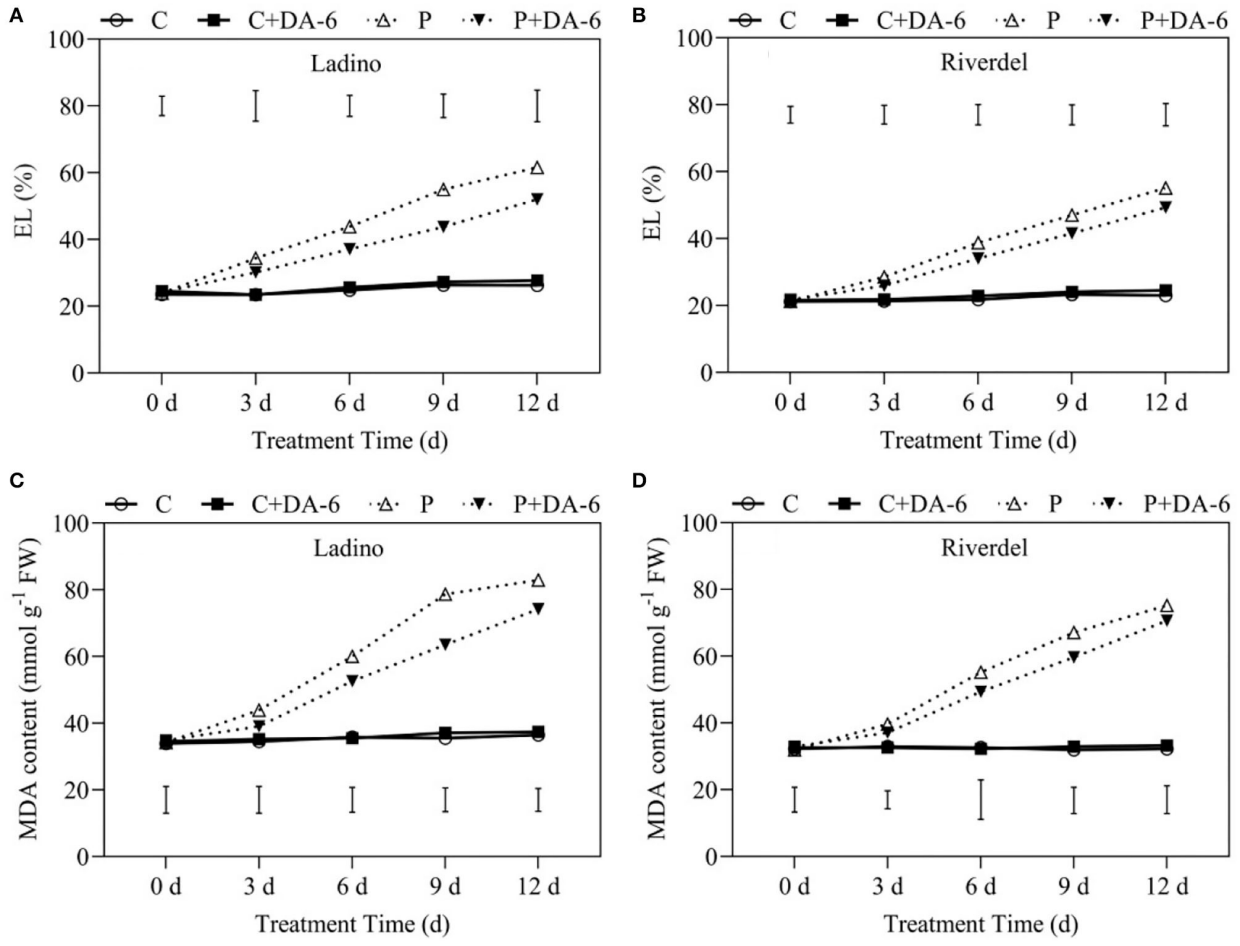
Effect of water-deficit stress and DA-6 pretreatment on **(A,B)** electrolyte leakage and **(C,D)** malondialdehyde (MDA) content in the two white clover cultivars (Ladino and Riverdel). Data were collected after 0, 3, 6, 9, and 12 days of water-deficit stress. Data are mean ± standard error (*n* = 4). Vertical bars demonstrate the LSD values (*p* ≤ 0.05). C, control treatment; C+DA-6, control + DA-6 pretreatment; P, water-deficit stress; P+DA-6, water-deficit stress + DA-6 pretreatment.

Leaf EL and MDA content associated with membrane damage exhibited same pattern in both cultivars when exposed to water-deficit stress and DA-6 treatment (Figures 3A–D). Both cultivars did not show significant difference in EL and MDA content under well-watered conditions; however, the DA-6-pretreated plants demonstrated marked reduction in EL level and MDA accumulation compared to the non-DA-6-treated plants at various intervals of water-deficit stress. In addition, the Ladino cultivar exhibited higher EL and MDA content than Riverdel in response to water-deficit stress (Figures 3A–D). Water-deficit stress resulted in considerable increases in EL level and MDA content; however, exogenous DA-6 pretreatment mitigated the water stress-induced intensification of EL and MDA in both cultivars. EL was reduced by 12.5, 15.4, 20.4, and 15.5% on days 3, 6, 9, and 12, respectively, of water-deficit stress in Ladino because of DA-6 treatment. In Riverdel, EL was reduced by 9.2, 12.1, 13.4, and 10.5%, respectively, on days 3, 6, 9, and 12 of water-deficit stress because of exogenous DA-6 application (Figures 3A,B). MDA content exhibited a similar trend like EL in the DA-6 pretreated plants when compared with the untreated plants under water-deficient conditions (Figures 3C,D).

### Effect of DA-6 Application on Leaf Lipidomic Changes in the Two White Clover Cultivars Under Water-Deficit Stress

**Figure 4:**
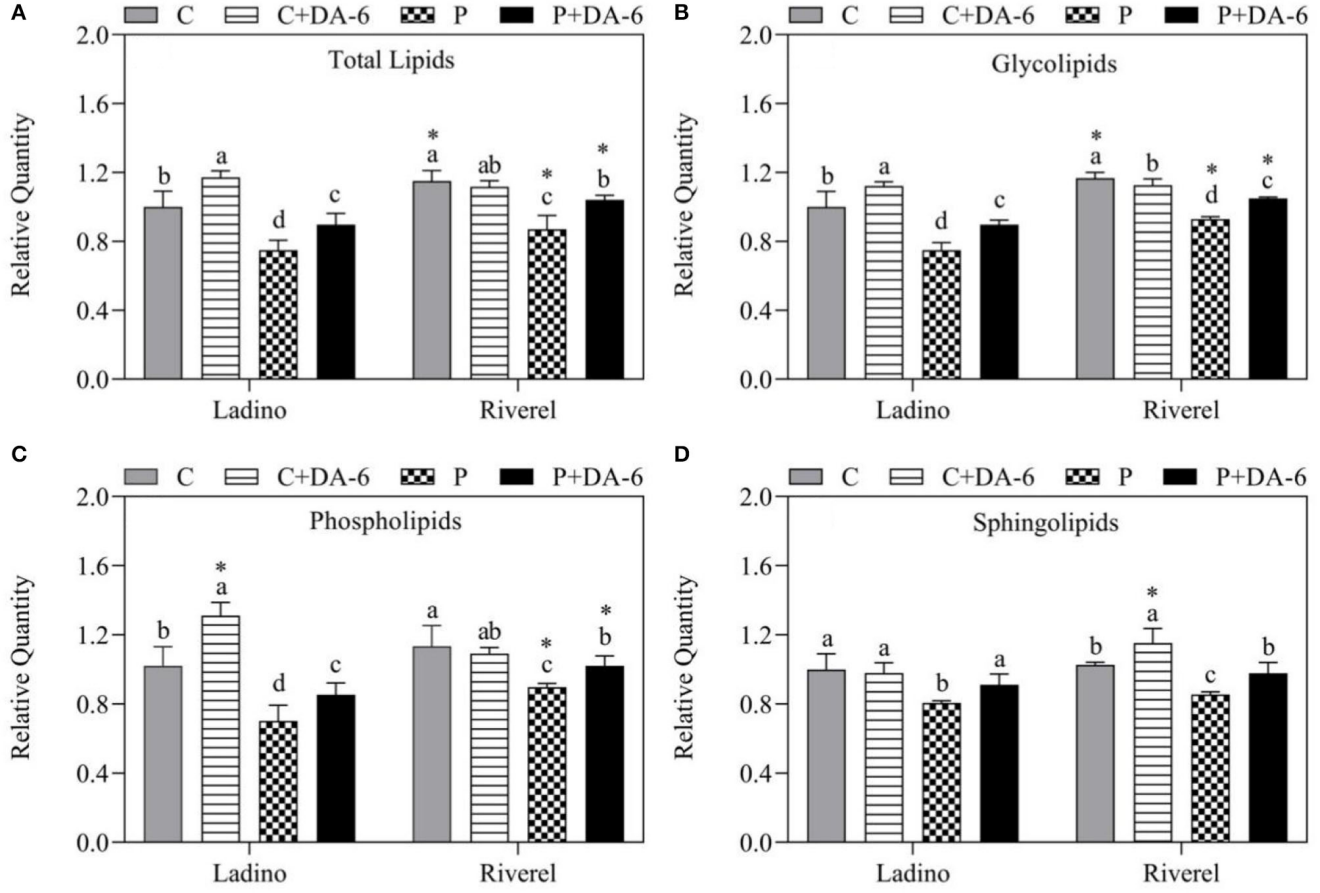
Effect of water-deficit-stress and DA-6 pretreatment **(A)** relative total lipid content, **(B)** glycolipid content, **(C)** phospholipid content, and **(D)** sphingolipid content in the two white clover cultivars (Ladino and Riverdel) after 9 days of water-deficit stress. Data are mean ± standard error (*n* = 4). Vertical bars demonstrate the LSD values (*p* ≤ 0.05). Different letters above the columns indicate significant differences between different treatments at a particular time duration under normal conditions or water stress. * represents the significant difference for a specific treatment (C, C+DA-6, P, or P+DA-6) between Ladino and Riverdel. C, control treatment; C+DA-6, control + DA-6 pretreatment; P, water-deficit stress; P+DA-6, water-deficit stress + DA-6 pretreatment.

Water-deficit stress significantly decreased the total lipid content of the two white clover cultivars, but the DA-6-pretreated Ladino and Riverdel exhibited significantly higher total lipid content than the untreated plants under water-deficit stress (Figure 4A). The Gll content in water-stress affected plants without DA-6 pretreatment was decreased by 25 and 19.8%, which is in contrast to well-watered Ladino or Riverdel plants, respectively (Figure 4B). Similarly, the Phl and Sph content was reduced by 31.1 and 20.7% and 19.3 and 16.6% in Ladino and Riverdel, respectively, under water-deficit stress (Figures 4C,D). Under normal conditions, foliar pretreatment with DA-6 of Ladino significantly enhanced the Gll and Phl content, but the Sph content remained unaffected (Figures 4B–D). In Riverdel, the exogenous application of DA-6 decreased the Gll content and increased the Sph content, but the Phl content remained unaffected compared with the control (Figures 4B–D). Under water-deficient conditions, the DA-6 pretreated plants exhibited a 16.7 and 16.4% increase in total lipid content in Ladino and Riverdel, respectively, compared to the untreated plants (Figure 4A). Exogenous DA-6 application significantly enhanced the Gll (16.6 and 11.3%) and Phl (17.6 and 12.1%) content in the two white clover cultivars (Ladino and Riverdel) under water-deficit stress (Figures 4B,C). Moreover, Spls were also increased by 11.4 and 12.5% in DA-6-treated Ladino and Riverel as compared to that in untreated plants exposed to water-deficit stress (Figure 4D).

**Figure 5:**
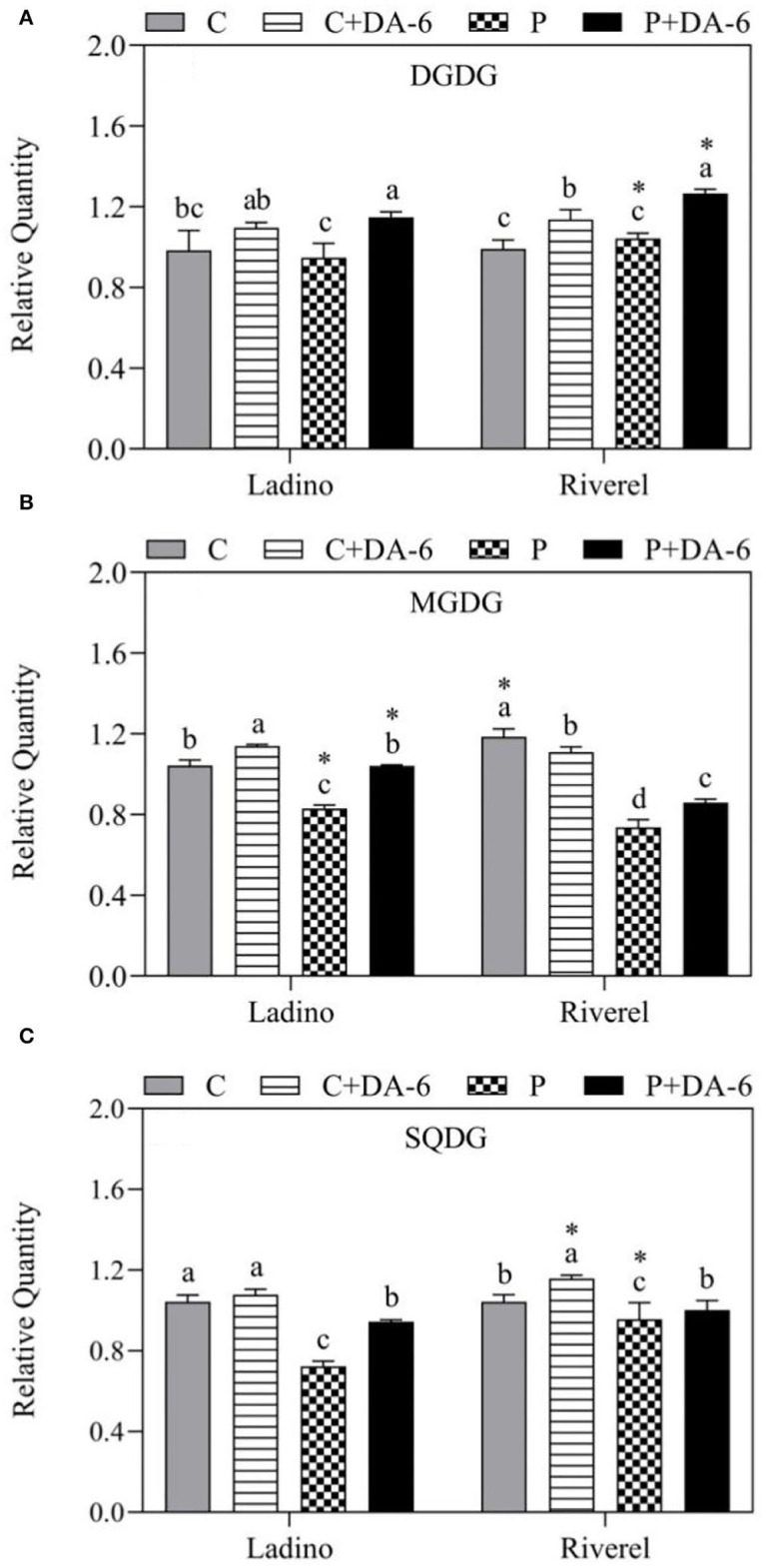
Effect of water-deficit stress and DA-6 pretreatment on relative content of **(A)** DGDG, **(B)** MGDG, and **(C)** SQDG in the two white clover cultivars (Ladino and Riverdel) after 9 days of water-deficit stress. Data are mean ± standard error (*n* = 4). Vertical bars demonstrate the LSD values (*p* ≤ 0.05). Different letters above the columns indicate significant differences between different treatments at a particular time duration under normal conditions or water stress. * represents the significant difference for a specific treatment (C, C+DA-6, P, or P+DA-6) between Ladino and Riverdel. C, control treatment; C+DA-6, control + DA-6 pretreatment; P, water-deficit stress; P+DA-6, water-deficit stress + DA-6 pretreatment; DGDG, digalactocyl diacylglycerol; MGDG, monogalactocyl-diacylglycerol; SQDG, sulfoquinovosyl diacylglycerol.

**Figure 6:**
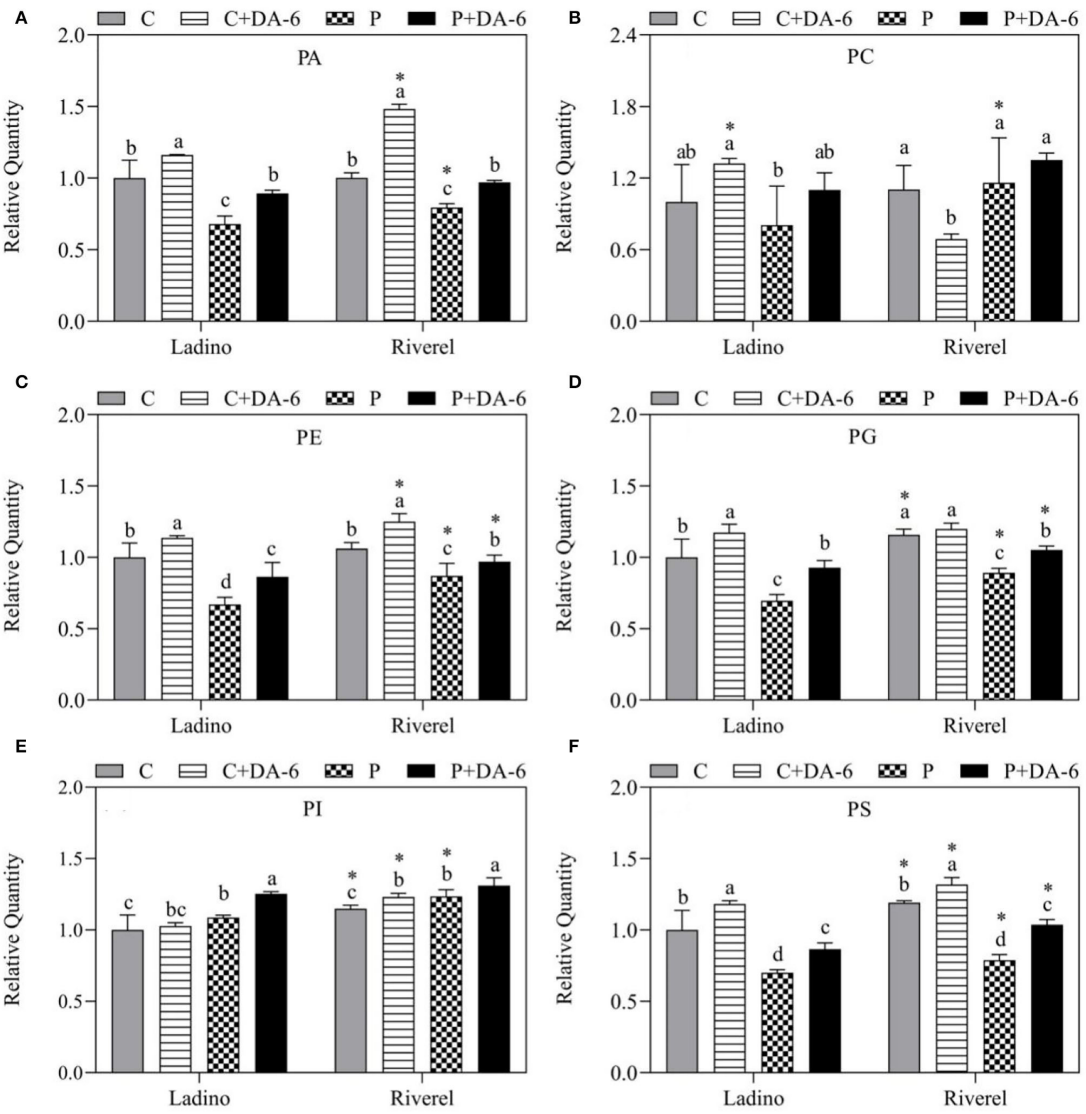
Effect of water-deficit stress and DA-6 pretreatment on relative content of **(A)** PA, **(B)** PC, **(C)** PE, **(D)** PG, **(E)** PI, and **(F)** PS in the two white clover cultivars (Ladino and Riverdel) after 9 days of water-deficit stress. Data are mean ± standard error (*n* = 4). Vertical bars demonstrate the LSD values (*p* ≤ 0.05). Different letters above the columns indicate significant differences between different treatments at a particular time duration under normal conditions or water stress. * represents the significant difference for a specific treatment (C, C+DA-6, P, or P+DA-6) between Ladino and Riverdel. C, control treatment; C+DA-6, control + DA-6 pretreatment; P, water-deficit stress; P+DA-6, water-deficit stress + DA-6 pretreatment; PA, phosphatidic acid; PC, phosphatidylcholine; PE, phosphatidylethanolamine; PG, phosphatidylglycerol; PI, phosphatidylinositol; PS, phosphatidylserine.

**Figure 7:**
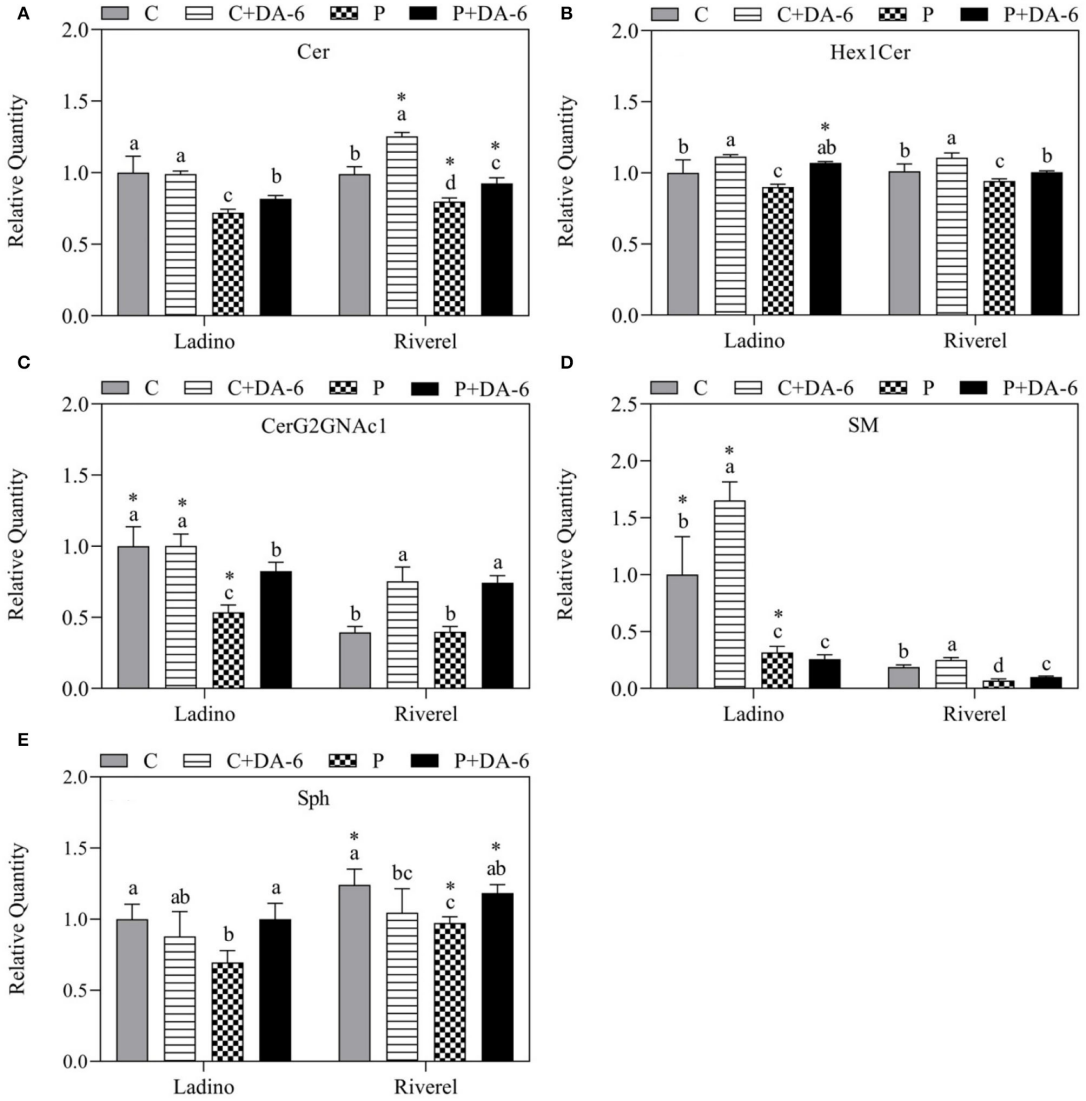
Effect of water-deficit stress and DA-6 pretreatment on relative content of **(A)** Cer, **(B)** Hex1Cer, **(C)** CerG2GNAc1, **(D)** SM, and **(E)** Sph in the two white clover cultivars (Ladino and Riverdel) after 9 days of water-deficit stress. Data are mean ± standard error (*n* = 4). The vertical bars demonstrate the LSD values (*p* ≤ .05). Different letters above the columns indicate significant differences between different treatments at a particular time duration under normal conditions or water stress. *represents the significant difference for a specific treatment (C, C+DA-6, P, or P+DA-6) between Ladino and Riverdel. C, control treatment; C+DA-6, control + DA-6 pretreatment; P, water-deficit stress; P+DA-6, water-deficit stress + DA-6 pretreatment; Cer, ceramide; Hex1Cer, hexosylmonoceramide (GlcCer); CerG2GAc1, neutral glycosphingolipids; SM, sphingomyelin; Sph, sphingosine.

Lipidomic analysis examined a total of 14 lipid classes in leaves of the two white clover cultivars including 3 Glls (MGDG, DGDG, and SQDG), 6 Phls (PA, PC, PE, PG, PI, and PS), and 5 Spls (Cer, Hex1Cer, CerG2GNAc1, SM, and Sph), as shown in Figures 5–7. Water-deficit stress significantly decreased the MGDG, SQDG, PA, PE, PG, PS, Cer, Hex1Cer, SM, and Sph content in both cultivars compared to the well-watered control, but the PI content was increased (Figures 5–7). In addition, water-deficit stress reduced the CerG2GNAc1 content in Ladino when compared to the non-stressed plants (Figure 7C). Exogenous application of DA-6 significantly enhanced the accumulation of DGDG (13.27 and 17.46%), MGDG (20.19 and 15.12%), SQDG (23.40 and 10.30%), PA (26.75 and 18.09%), PE (22.46 and 10.36%), PG (25.05 and 15.29%), PI (13.29 and 5.68%), PS (19 and 23.99%), Cer (8.25 and 13.85%), Hex1Cer (15.8 and 4.89%), CerG2GNAc1 (35.04 and 46.25%), and Sph (30.38 and 17.74%) in both cultivars (Ladino and Riverdel) under water-deficit stress (Figures 5–7). In addition, a significant increase in SM content was observed in the DA-6 treated Riverdel plants exposed to water-deficit stress, but there was no substantial effect on Ladino (Figure 7D). PC content did not show any significant difference between DA-6-treated and untreated plants subjected to water stress in both cultivars (Figure 6B).

**Figure 8:**
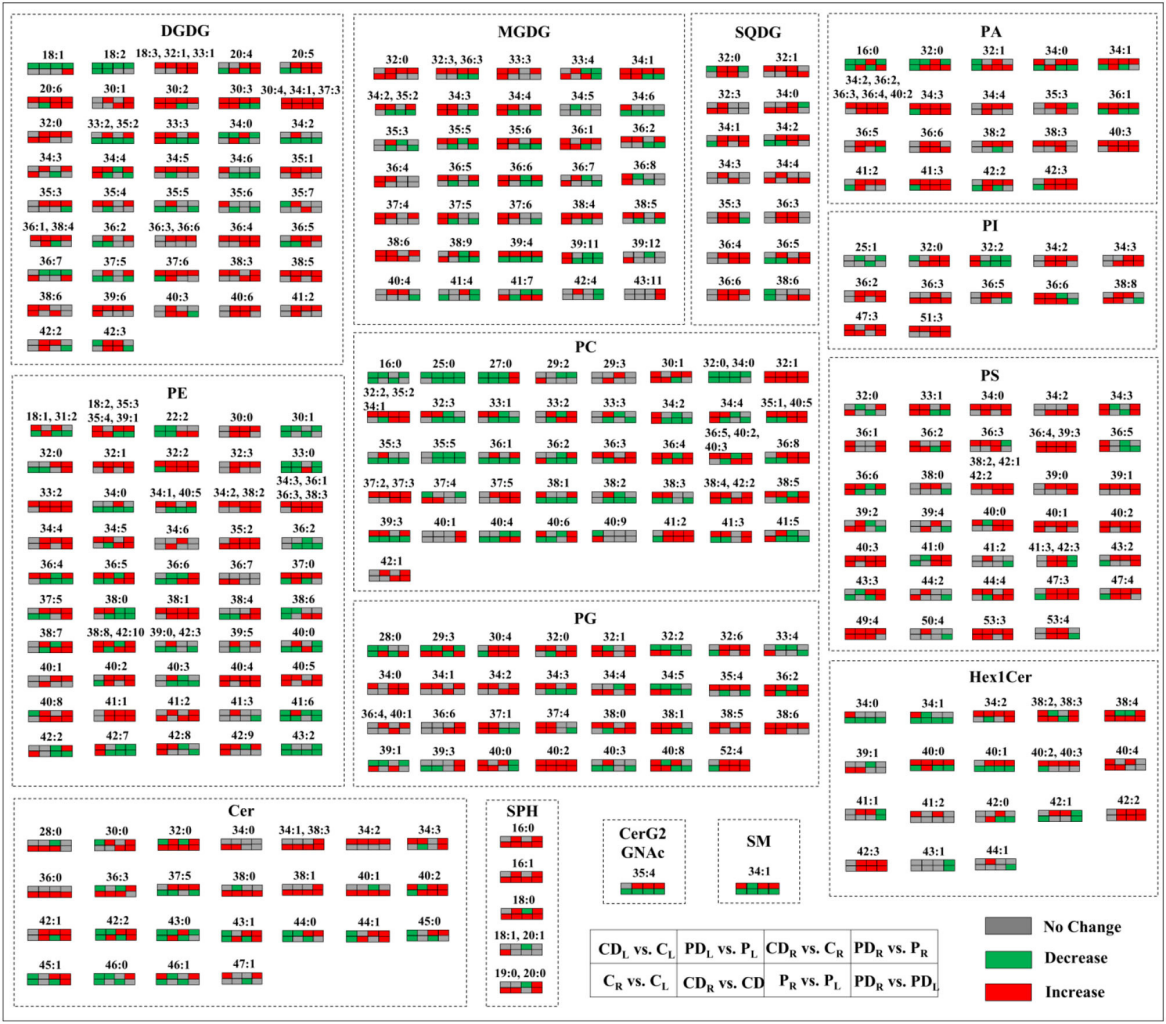
Comparison between various treatments with or without DA-6 pretreatment in terms of different lipid molecular species in the two white clover genotypes (Ladino and Riverdel) under normal and water-deficient conditions. C_L_, Ladino grown under normal conditions; CD_L_, DA-6-pretreated Ladino grown under normal conditions; P_L_, Ladino grown under water-limited conditions; PD_L_, DA-6-pretreated Ladino grown under water-limited conditions; C_R_, Riverdel grown under normal conditions; CD_R_, DA-6-pretreated Riverdel grown under normal conditions; P_R_, Riverdel grown under water-limited conditions; PD_R_, DA-6-pretreated Riverdel grown under water-limited conditions.

Water-deficit stress or water-deficit stress in combination with DA-6 application significantly altered lipid molecular species in the two cultivars under normal and water-deficient conditions, as shown in Figure 8. For Gll molecular species, both DA-6-treated cultivars showed significantly higher DGDG molecular species (18:3, 20:5, 20:6, 30:1, 30:2, 30:3, 30:4, 32:0, 32:1, 33:1, 33:2, 33:3, 34:1, 34:3, 34:4, 34:5, 35:1, 35:2, 35:3, 35:4, 36:1, 36:2, 36:3, 36:4, 36:5, 36:6, 37:3, 37:5, 37:6, 38:3, 38:4, 38:5, 39:6, 40:6, and 41:2), MGDG molecular species (32:0, 32:3, 33:3, 34:1, 34:2, 34:3, 34:4, 35:2, 35:5, 35:6, 36:2, 36:3, 36:5, 36:6, 37:4, 38:4, 38:5, 38:6, 38:9, and 39:4), and SQDG molecular species (34:1, 34:2, 36:4, 36:5, and 36:6) when compared with the untreated plants under water-deficit stress (Figure 8).Various Phl molecular species, such as PA (34:1, 34:2, 34:3, 34:4, 36:1, 36:2, 36:3, 36:4, 36:5, 38:2, 40:2, 40:3, 41:2, 41:3, 42:2, and 42:3), PC (32:1, 32:2, 34:1, 35:1, 35:2, 36:1, 36:3, 36:4, 36:5, 36:8, 37:2, 37:3, 37:5, 38:4, 38:5, 39:3, 40:2, 40:3, 40:5, 41:2, 41:3, and 42:2), PE (30:0, 32:1, 32:2, 32:3, 33:2, 34:1, 34:2, 34:3, 34:4, 34:5, 35:2, 36:1, 36:3, 36:4, 36:5, 37:0, 37:5, 38:1, 38:2, 38:3, 38:7, 38:8, 39:5, 40:2, 40:4, 40:5, 40:8, 41:1, 41:2, 42:9, and 42:10), PG (30:4, 32:0, 32:6, 34:1, 34:2, 34:3, 35:4, 36:2, 36:4, 38:0, 38:5, 38:6, 40:1, 40:2, and 52:4) PI (34:2, 36:2, 36:5, and 51:3), and PS (33:1, 34:0, 36:4, 36:6, 38:2, 39:0, 39:1, 39:3, 40:1, 40:2, 40:3, 42:1, 42:2, 43:2, 44:4, 47:3, 47:4, 49:4, and 53:3), were significantly enhanced by DA-6 application in both cultivars subjected to water-deficient conditions (Figure 8). For Spl molecular species, DA-6-treated plants demonstrated significant improvement in the Cer (30:0, 32:0, 34:2, 34:3, 37:5, or 42:1), Hex1Cer (34:2, 40:0, 40:1, 40:2, 40:3, 42:2, or 42:3), CerG2GNAc1 (35:4), and Sph (16:0, 16:1 or 18:0) in two cultivars but only SM (34:1) in Riverdel than untreated plants subjected to water stress (Figure 8).

### Effect of DA-6 Application on Ratio and Unsaturation Index of Lipids in Two White Clover Cultivars Under Water-Deficit Stress

**Figure 9:**
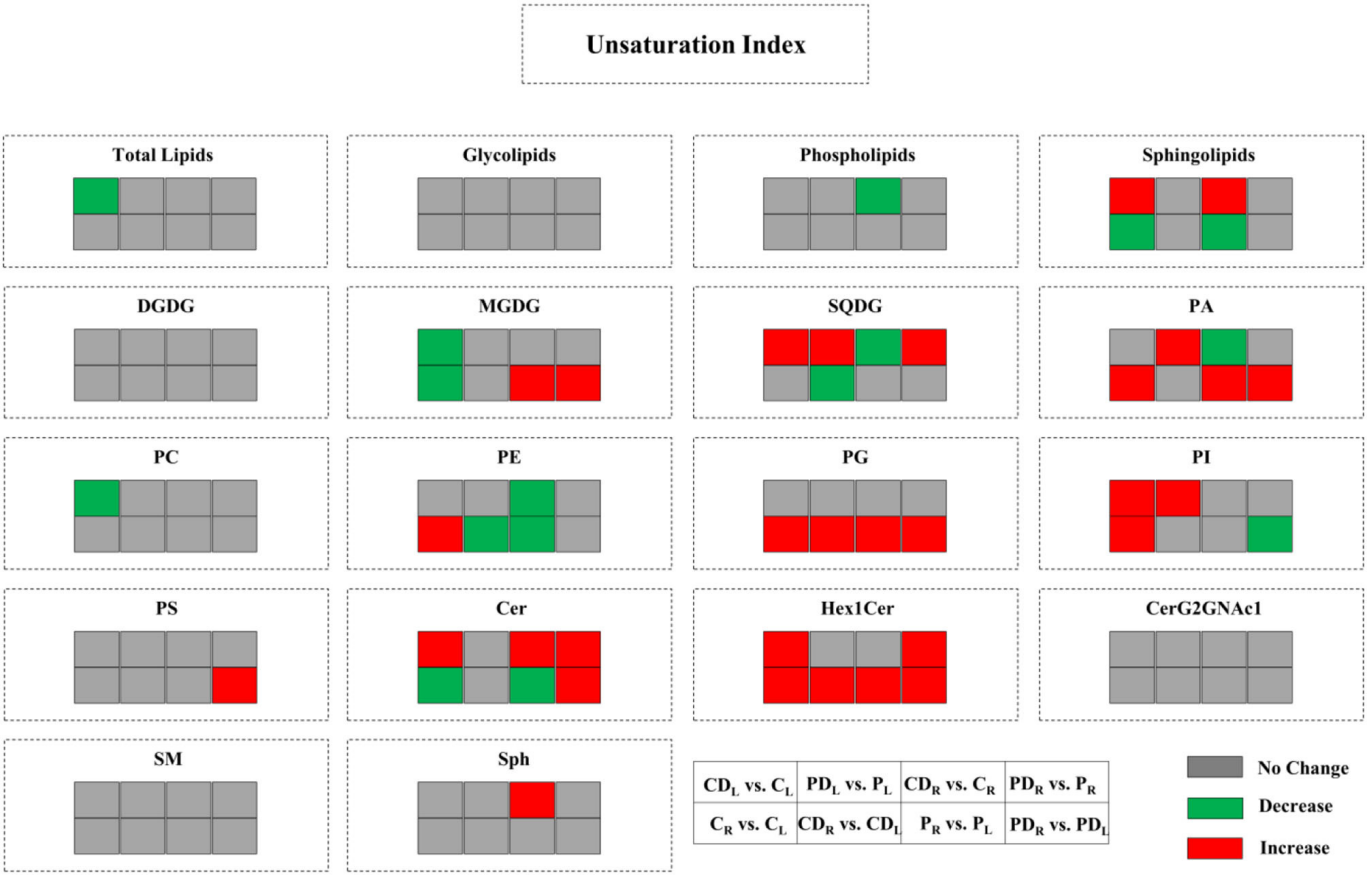
Effect of water-deficit stress and DA-6 pretreatment on unsaturation index of total lipids, glycolipids, phospholipids, sphingolipids, DGDG, MGDG, SQDG, PA, PC, PE, PG, PI, PS, Cer, Hex1Cer, CerG2GNAc1, SM, and Sph in the two white clover cultivars (Ladino and Riverdel) after 9 days of water-deficit stress. C_L_, Ladino grown under normal conditions; CD_L_, DA-6-pretreated Ladino grown under normal conditions; P_L_, Ladino grown under water-limited conditions; PD_L_, DA-6-pretreated Ladino grown under water-limited conditions; C_R_, Riverdel grown under normal conditions; CD_R_, DA-6-pretreated Riverdel grown under normal conditions; P_R_, Riverdel grown under water-limited conditions; PD_R_, DA-6-pretreated Riverdel grown under water-limited conditions.

Water-deficit stress significantly improved the DGDG:MGDG ratio in both cultivars when compared to the well-watered control (Table 1). The DA-6-treated Riverdel exhibited significantly higher DGDG:MGDG ratio than the untreated plants under water-deficit stress (Table 1). Water-deficit stressed Riverdel with DA-6 application also showed higher PC:PE ratio in contrast to the well-watered control plants (Table 1).Under water-deficit stress, the unsaturation index of DGDG, PS, Cer, Hex1Cer, CerG2GNAc1, SM, and Sph in the two cultivars did not exhibit significant difference in the stressed plants without DA-6 application when compared to the well-watered control (Figure 9). The unsaturation index of MGDG, PA, PC, and PE in Ladino remained unaffected by water stress. Water-deficit stress significantly reduced the unsaturation index of SQDG, PG, and PI in both cultivars (Figure 9). In addition, the unsaturation index of PE also decreased in Riverdel compared to the control under water-deficit stress. In Riverdel, water-deficit affected plants without DA-6 treatment exhibited a significant increase in the unsaturation index of MGDG, PA, and PC, which is in contrast to the well-watered control (Figure 9). In both cultivars, DA-6 application significantly increased the unsaturation index of SQDG compared to the untreated plants under water stress. The foliar treatment of DA-6 increased the unsaturation index of PA and PI in Ladino and Cer and Hex1Cer in Riverdel when compared to the untreated plants under water-deficient conditions (Figure 9).

**Table 1:** DGDG:MGDG and PC:PE ratio in Ladino and Riverdel with or without DA-6 pre-treatment under water-deficit stress.

**Ratio**	**C**	**C+D**	**P**	**P+D**
**Ladino**				
DGDG:MGDG	0.997 ± 0.026 b	0.975 ± 0.013 b	1.094 ± 0.046 a	1.101 ± 0.026 a
PC:PE	0.991 ± 0.274 a	1.164 ± 0.032 a	1.200 ± 0.470 a	1.275 ± 0.071 a
**Riverdel**				
DGDG:MGDG	0.835 ± 0.020 d	1.013 ± 0.010 c	1.416 ± 0.048 b	1.471 ± 0.011 a
PC:PE	1.043 ± 0.193 b	0.553 ± 0.033 c	1.316 ± 0.341 ab	1.394 ± 0.109 a

*C, control treatment; C+DA-6, control + DA-6 pretreatment; P, water-deficit stress; P+DA-6, water-deficit stress + DA-6 pretreatment; DGDG, digalactocyl diacylglycerol; MGDG, monogalactocyl-diacylglycerol; PC, phosphatidylcholine; PE, phosphatidylethanolamine*.

## Discussion

Plants have evolved various adaptive and tolerance mechanisms to survive water-deficit stress. Drought resistance differs greatly from plant species to species or genotype to genotype in nature, which provides vital basic materials for exploration of stress mechanism and breeding for stress resistance (Li et al., 2013; Basu et al., 2016). Our present study revealed that physiological responses including leaf water status and utilization (RWC, OP, and WUE), photosynthetic performance (Chl content, Fv/Fm, PIABS, and Pn), and oxidative damage and membrane stability (MDA and EL) exhibited significant differences in the two white clover cultivars, with Riverdel demonstrating better drought resistance than Ladino (Figures 1–3). Similar findings have been shown in our previous studies. Drought-resistant white clover genotypes had significantly higher osmotic adjustment and membrane stability as well as less oxidative damage than drought-susceptible ones when subjected to same duration of water-deficit stress (Li et al., 2013, 2015). Although both cultivars (Riverdel and Ladino) suffered from severe water-deficit damage, the exogenous application of DA-6 could effectively alleviate water deficit-induced damaging effects on the two cultivars during day 12 of water scarcity (Figures 1–3). A previous study has also shown that DA-6 application enhanced the tolerance of *Cassia obtusifolia* to salt stress by maintenance of better PSII and antioxidant capacity (Zhang et al., 2016). Moreover, DA-6 pretreatment mitigated cold-induced oxidative damage by improving the antioxidant defense system and photosynthesis in strawberry (*Fragaria ananassa*) seedlings (Fu et al., 2011). Our findings indicated positive effects of DA-6 on regulating amelioration in drought resistance of both white clover cultivars associated with enhanced water homeostasis, photosynthetic performance, and cell membrane stability. How DA-6-induced reprogramming of lipid profiling affected membrane stability and photosynthetic performance during water-deficit stress was discussed in detail below.

Previous studies have reported that abiotic stresses restrained lipids biosynthesis as shown by decreased total lipids and Gll, Phl, and Spl contents in many plant species such as *Lolium festuca* (Perlikowski et al., 2016) under water-deficit stress, seashore paspalum (Gao et al., 2020) under salt stress, tall fescue (Zhang et al., 2019) under heat stress, and *Arabidopsis* (Nagano et al., 2014) under cold stress. Water-deficit stress decreased the total lipids and Gll, Phl, and Spl contents in both white clover cultivars, while the downtrend of these lipids was less in Riverdel than in Ladino. However, exogenous supply of DA-6 significantly alleviated water deficit-induced declines in total lipids and Gll, Phl, and Spl contents in both white clover cultivars (Figure 4). Similar findings were demonstrated in the study of Gao et al. (2020) who noticed that significant increases in total lipid content and Gll and Phl contents through exogenous choline treatment were propitious to maintain membrane structure and functionality in seashore paspalum plants under salt stress. According to the present findings, DA-6 played a key role in maintaining lipid synthesis in the two white clover cultivars subjected to water-deficit stress, which could contribute toward ameliorated drought resistance associated with membrane integrity, stability, and fluidity.

Glls including DGDG, MGDG, and SQDG are primary constituents in thylakoid membranes and envelope of chloroplasts, hence playing critical functions in photosynthesis (Frentzen, 2004; Sato, 2004; Hölzl et al., 2009; Kobayashi, 2016; Fujii et al., 2017, 2018). The equilibrium between bilayer DGDG and non-bilayer MGDG and adequate proportion of SQDG and PG in chloroplastic membranes are essential to sustain membrane stability and efficiency of vital proteins involved in the process of photosynthesis (Páli et al., 2003; Hölzl and Dörmann, 2007). It has been shown that reduction in Gll content leads to Chl degradation and a decrease in photosynthetic function (Dörmann and Benning, 2002). Water-deficit stress caused a decline in the MGDG content of wheat leaves in the seedling stage (Wang et al., 2020). Furthermore, a study of Gasulla et al. (2013) illustrated that water-deficit stress induced a substantial decrease in the SQDG content of drought-sensitive *Craterostigma plantagineum* cultivars when compared to tolerant ones. MGDG and SQDG contents declined significantly in both white clover cultivars exposed to water-deficit stress; however, DGDG remained unaffected under water-deficit stress (Figure 5). The DA-6 application enhanced the accumulation of DGDG (18:3, 20:5, 20:6, 30:1, 30:2, 30:3, 30:4, 32:0, 32:1, 33:1, 33:2, 33:3, 34:1, 34:3, 34:4, 34:5, 35:1, 35:2, 35:3, 35:4, 36:1, 36:2, 36:3, 36:4, 36:5, 36:6, 37:3, 37:5, 37:6, 38:3, 38:4, 38:5, 39:6, 40:6, and 41:2) under water-deficit stress and alleviated water stress-induced declines in MGDG (32:0, 32:3, 33:3, 34:1, 34:2, 34:3, 34:4, 35:2, 35:5, 35:6, 36:2, 36:3, 36:5, 36:6, 37:4, 38:4, 38:5, 38:6, 38:9, and 39:4) and SQDG (34:1, 34:2, 36:4, 36:5, and 36:6) contents in both cultivars (Figures 5, 8). Drought-priming increased DGDG (36:6) and MGDG (34:4, 36:6) contents in leaves of tall fescue (*Festuca arundinacea*), which contributed to better stability and integrity of thylakoid membranes when tall fescue suffered from sustained heat stress (Zhang et al., 2019). In addition, significantly higher DGDG:MGDG ratio was noticed in Riverdel pretreated with DA-6 under water-deficit stress (Table 1). It has been reported that higher DGDG:MGDG ratio in membranes is not only important for the folding and insertion of proteins (Bogdanov and Dowhan, 1999; Dörmann and Benning, 2002) but is also a key indicator of better bilayer structure associated with maintenance of chloroplast membrane stability and fluidity in plants under water-deficit stress(Torres-Franklin et al., 2007; Chen et al., 2018a). In addition to Glls, PG is another lipid class located in thylakoid membranes and plays an important role in proper chloroplast function (Wang et al., 2010). Various studies have found that PG content was decreased significantly in plants under abiotic stresses such as salt and water scarcity (Guerfel et al., 2008; Gao et al., 2020). Improved thermotolerance induced by drought priming was associated with PG (34:3 and 34:4) accumulation in tall fescue (Zhang et al., 2019). In our study, PG content was decreased substantially in the two white clover cultivars under water-deficit stress. However, the DA-6 pretreatment significantly improved PG (30:4, 32:0, 32:6, 34:1, 34:2, 34:3, 35:4, 36:2, 36:4, 38:0, 38:5, 38:6, 40:1, 40:2, and 52:4) content in both cultivars (Figures 6, 8). Physiological responses also showed that water deficit-caused photoinhibition and decline in photochemical efficiency could be alleviated significantly by the application of DA-6 (Figure 2). These results proposed that improved drought resistance in white clover cultivars could be the result of improved DGDG:MGDG ratio and maintenance of PG and Gll (DGDG, MGDG, and SQDG) contents responsible for higher thylakoid membrane stability and functionality associated with better maintenance of photosynthesis under water-deficient conditions.

PC and PE, two of the most abundant Phl species, are available in non-chloroplastic membranes for maintenance of membrane structure and function in plants (Larsson et al., 2006). PC acts as a precursor in the synthesis of various Glls (Ohlrogge and Browse, 1995), and PE is highly involved in signaling pathways under stress conditions (Chapman, 2000). A significant decrease in PE content has been observed in the leaves of olive trees subjected to water-deficit stress (Guerfel et al., 2008). The present findings revealed that water-deficit stress decreased PE content in the two white clover cultivars when compared to the respective controls, but PC content did not exhibit marked difference in both white clover cultivars with or without DA-6 treatment under water-deficit stress (Figure 6). Both DA-6-pretreated cultivars demonstrated higher contents of PE (30:0, 32:1, 32:2, 32:3, 33:2, 34:1, 34:2, 34:3, 34:4, 34:5, 35:2, 36:1, 36:3, 36:4, 36:5, 37:0, 37:5, 38:1, 38:2, 38:3, 38:7, 38:8, 39:5, 40:2, 40:4, 40:5, 40:8, 41:1, 41:2, 42:9, and 42:10) and PC (32:1, 32:2, 34:1, 35:1, 35:2, 36:1, 36:3, 36:4, 36:5, 36:8, 37:2, 37:3, 37:5, 38:4, 38:5, 39:3, 40:2, 40:3, 40:5, 41:2, 41:3, and 42:2) molecular species in contrast to the untreated plants under water-deficit stress (Figure 8). Higher levels of PE in the plasma membrane of plants exposed to water stress could help to alleviate membrane damage in wilting protoplasts and maintain catalytic activities of membrane enzymes through enhanced lipid-protein interactions (Larsson et al., 2006). A previous study conducted on grapevines also illustrated that water deficit induced a significant increase in PC/PE ratio in cell membranes (Toumi et al., 2008). Higher PC/PE ratio was linked with enhanced membrane stability, fluidity, and flexibility because of the bilayer-prone characteristic of PC (Narayanan et al., 2018). This study showed higher PC/PE ratio in Riverdel than in Ladino under water-deficit stress. In addition, significantly higher PC/PE ratio was only observed in the water deficit-stressed Riverdel pretreated with DA-6 when compared with the well-watered control (Table 1). These results indicated that the higher PC:PE ratio in Riverdel is an important reason for enhanced membrane stability and better adaption to water-deficit stress compared with Ladino. The DA-6-regulated drought resistance was related to alterations in molecular species of PC and PE.

PI is an imperative constituent of the plasma membrane and is involved in the regulation of signaling pathways and maintenance of cell metabolic activities under adverse environmental conditions. It acts as a critical precursor in biosynthesis pathways of various Phls, such as PIP, PIP2, and PA, which initiate signal transduction to trigger specific genes in relation to plant adaption to environmental hazards (Löfke et al., 2008; Xue et al., 2009). Among Phls, PA is a well-known secondary messenger that performs regulatory functions in lipid metabolism, signaling transduction pathways (e.g., calcium and ROS signaling), and cytoskeleton dynamics (Testerink and Munnik, 2005; Zhao, 2015). Earlier studies have revealed that PLD-synthesized PA was involved in adaptive responses to cold stress (Li et al., 2004), heat stress (Wang and Huang, 2017), and water-deficit stress (Chen et al., 2018b) in different plant species. *Phosphatidylinositol synthase* (*PIS*) gene overexpression in *Nicotiana tobaccum* enhanced PI and other Phls as well as Glls (PE, PC, PG, DGDG, and MGDG) accumulation associated with improved membrane stability and integrity under water-deficit stress (Zhai et al., 2012). Our current study showed that DA-6 not only further promoted water deficit-induced PI accumulation but also significantly alleviated water deficit-induced decline in PA in both cultivars (Figure 6). These findings indicated that ameliorated resistance by DA-6 and better drought resistance in Riverdel could be associated with maintenance of PI and PA synthesis contributing to enhanced membrane stability, fluidity, and structural integrity. For changes in different molecular species of PI and PA, the DA-6 pretreatment significantly enhanced the PA (34:1, 34:2, 34:3, 34:4, 36:1, 36:2, 36:3, 36:4, 36:5, 38:2, 40:2, 40:3, 41:2, 41:3, 42:2, and 42:3) and PI (34:2, 36:2, 36:5, and 51:3) content in both cultivars in response to water-deficit stress (Figure 8). It has been found that significant increases in PA (34:1, 34:2, and 36:5) and PI (34:3 and 34:4) induced by choline pretreatment and drought priming were beneficial for plants to acclimate to salt stress or heat stress (Zhang et al., 2019; Gao et al., 2020).

PS species are confined to the interior surface of cell membranes, and their roles are not well-documented in plants as other Phls (Vance and Steenbergen, 2005). It is worth mentioning that PS is involved in the biosynthesis of other Phl species such as PC and PE (Larsson et al., 2006). Long-chain fatty acids consisting of molecular species of PS could facilitate cellular transport and maintenance of the bilayer curvature in the plasma membrane (Vincent et al., 2001). The study of Gasulla et al. (2013) found that PS content was increased significantly in the cell membrane of desiccation-tolerant *Craterostigma plantagineum* but was significantly decreased in two desiccation-sensitive species (*Lindernia subracemosa* and *Arabidopsis thaliana*) in response to water-deficit stress, indicating that enhanced PS accumulation is in favor of better adaptation to water stress. Although water-deficit stress significantly decreased the PS content in both white clover cultivars, Riverdel accumulated more PS content than Ladino under well-watered and water-limited conditions, which could be one of the important reasons for better stress resistance and genetic superiority of Riverdel under water-deficient conditions. In addition, the application of DA-6 improved PS accumulation under well-watered conditions and effectively alleviated water deficit-induced decline in the PS content of both of Riverdel and Ladino. However, the function and the role of DA-6-induced increases in PS molecular species (33:1, 34:0, 36:4, 36:6, 38:2, 39:0, 39:1, 39:3, 40:1, 40:2, 40:3, 42:1, 42:2, 43:2, 44:4, 47:3, 47:4, 49:4 or 53:3) in Riverdel and Ladino needs to be further investigated in white clover under water-deficit stress (Figure 8).

Spls are structural components of lipid bilayers and perform their roles *via* bioactive metabolites as mediators of primary vesicular and physiological functions in plants. In addition, Spls are also involved in signal transduction under unfavorable environmental conditions (Michaelson et al., 2016). For instance, the ceramide constituent of Spls has been reported to be involved in the regulation of programmed cell death (PCD) (Liang et al., 2003). Spls play a vital role as signaling molecules during ABA-regulated guard cell closure (Ng et al., 2001; Coursol et al., 2003, 2005). Cer acts as the backbone of Spls and functions as a key signaling molecule to regulate stress signal transduction in plants (Hanada, 2003; Orešič et al., 2008). GlcCers (e.g., Hex1Cer), also known as glycosylceramides, are one of the highly abundant Spl groups in the plasma membrane, tonoplast of plant cells, and envelope of chloroplast membranes and take part in the maintenance of membranes stability (Lynch and Steponkus, 1987; Cahoon and Lynch, 1991; Uemura and Steponkus, 1994; Uemura et al., 1995; Spassieva and Hille, 2003). Previous studies have shown that GlcCer content was reduced to one-half in cell membranes during cold stress in rye (*Secale cereale*) and *Arabidopsis* (Lynch and Steponkus, 1987; Uemura and Steponkus, 1994; Uemura et al., 1995; Nagano et al., 2014). In this study, Sph, Cer, and Hex1Cer were significantly decreased in both white clover cultivars under water-deficit stress. However, the exogenous application of DA-6 increased the Cer (30:0, 32:0, 34:2, 34:3, 37:5, and 42:1), Hex1Cer (34:2, 40:0, 40:1, 40:2, 40:3, 42:2, and 42:3), and Sph (16:0, 16:1, and 18:0) contents in the two white clover cultivars under water-deficit stress, which could be associated with DA-6-regulated drought resistance by maintenance of membrane structure, chloroplast functionality, and signaling transduction pathways. In addition, SM and neutral glycosphingolipids (e.g., CerG2GNAc1) are the major Spl groups in animals and are available in minute quantities in plants, but their potential roles in plants have not been well-revealed and discussed in detail (Lester and Dickson, 1993; Geta Tafesse et al., 2006). Water-deficit stress significantly reduced SM in both cultivars and decreased the CerG2GNAc1 content in Ladino but did not affect the CerG2GNAc1 content in Riverdel (Figure 7). However, whether differential responses of CerG2GNAc1 and SM contributed to different stress resistance between Riverdel and Ladino or whether DA-6 improving CerG2GNAc1 and SM accumulation proved beneficial for drought resistance needs to be further investigated in plants, since little information is available on their roles in plants under environmental stresses so far.

Significant alteration in the degree of unsaturation of membrane lipids is an important adaption approach governed by fatty acid desaturases under unfavorable environmental conditions (Upchurch, 2008; Tshabuse et al., 2018). Unsaturation index has been widely used as an indicator of the degree of unsaturation. The higher unsaturation index of various lipids showed a positive correlation with better membrane stability and fluidity when plants suffer from a long period of water-deficit stress (Quartacci et al., 2002; Gigon et al., 2004; Toumi et al., 2008). Our results revealed that the unsaturation indexes of PA and PC in Riverdel were increased under water-deficit stress and that the unsaturation index of PE was decreased significantly in response to water-deficit stress (Figure 9). In addition, water-deficit stress significantly reduced the unsaturation index of SQDG, PG, and PI in both white clover cultivars. The exogenous application of DA-6 significantly increased the unsaturation index of PA and PI in Ladino and Cer and Hex1Cer in Riverdel under water-deficit stress (Figure 9). Moreover, DA-6 also improved the unsaturation index of SQDG in both cultivars under water-deficit stress (Figure 9). These results indicated that the DA-6 regulated resistance in Ladino and Riverdel could be associated with improved unsaturation index of the SQDG, PG, PI, Cer, and Hex1Cer classes contributing to enhanced membrane fluidity and integrity under water-deficit stress.

## Conclusions

**Figure 10:**
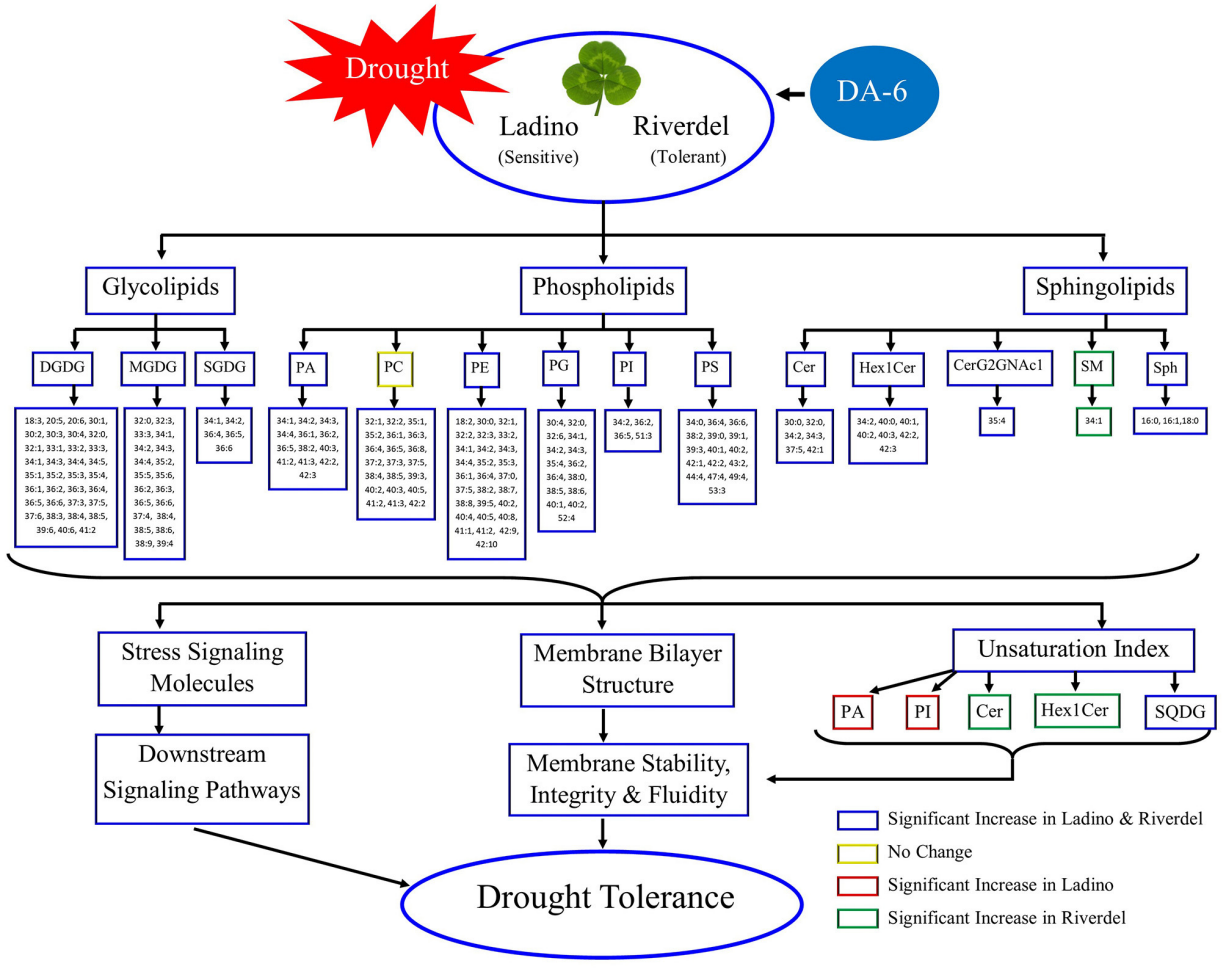
A comprehensive diagram of lipid remodeling and subsequent responses mediated by DA-6 pretreatment in the two white clover genotypes under water-deficit stress.

Water-deficit stress induced a significant increase in oxidative damage and a decline in the relative content of Glls, Phls, and Spls associated with reduced photosynthetic performance in the two white clover genotypes (Ladino and Riverdel) differing in drought resistance. Genotypic differences in drought resistance of the two genotypes and the beneficial effects of DA-6 pretreatment were correlated with changes in lipidomic profiling, unsaturation index, and ratio of lipids (Figure 10). A possible mechanism of drought resistance linked with lipid metabolism was that drought-resistant Riverdel could maintain higher lipid content, DGDG/MGDG ratio, and PC/PE ratio than drought-sensitive Ladino during the same duration of water-deficit stress. The DA-6 pretreatment induced significant increases in DGDG, MGDG, SQDG, PA, PE, PG, PI, PS, Cer, Hex1Cer, CerG2GNAc1 and Sph contents in both white clover cultivars. DA-6 also increased the DGDG:MGDG ratio and the relative content of SM in drought-resistant Riverdel. In addition, DA-6 increased the unsaturation index of PA and PI in Ladino and of Cer and Hex1Cer in Riverdel as well as the SQDG content in both cultivars under water-deficit stress. The DA-6-regulated lipid reprogramming and improvement of DGDG:MGDG ratio and unsaturation index of lipid species conferred enhanced membrane stability, integrity, fluidity, and downstream signaling transduction when white clover suffered from water-deficit stress.

## Data Availability Statement

The raw data supporting the conclusions of this article will be made available by the authors, without undue reservation.

## Author Contributions

ZL conceived and designed the research. MH, HQ, and SH conducted the experiments. MH, HQ, and BC evaluated the data. ZL and YP provided the different chemical reagents and experimental materials. Article writing was completed by MH and ZL. WL, GF, and JZ reviewed and edited the manuscript. All authors contributed to the article and approved the submitted version.

## Funding

This research was supported by the Sichuan Forage Innovation Team Project of the Industrial System Construction of Modern Agriculture of China (sccxtd-2020-16).

## Conflict of Interest

The authors declare that the research was conducted in the absence of any commercial or financial relationships that could be construed as a potential conflict of interest.

## Publisher's Note

All claims expressed in this article are solely those of the authors and do not necessarily represent those of their affiliated organizations, or those of the publisher, the editors and the reviewers. Any product that may be evaluated in this article, or claim that may be made by its manufacturer, is not guaranteed or endorsed by the publisher.
